# Supplementary material for: miR-146a, miR-221, and miR-155 are Involved in Inflammatory Immune Response in Severe COVID-19 Patients
Source: Diagnostics (Basel). 2022 Dec 30;13(1):133. doi: 10.3390/diagnostics13010133 (PMC9818442; doi:10.3390/diagnostics13010133)
Supplement: Supplementary file 1 [file diagnostics-13-00133-s001.zip › diagnostics-2070605-supplementary - references.pdf]

Supplementary Table 1. miRNAs selection criteria

miRNAs	Justification for validation	Reference
miR-16	Involved in regulation of inflammation and programmed cell death, acute lung injury, sepsis, phenotypic changes on T cells survival, differentiation, and proliferation.<br><br>Targeting genes responsible for host-SARS-CoV-2 interaction. Influence viral entry receptor (ACE2) related networks	[19,35,44,57,77–80]<br><br>[57,80,81]
miR-155	Involved in inflammation. T cell differentiation and innate immunity.<br><br>Regulates pathways related with IFN superfamily, NF-kB and MAP Kinase pathways. Cardiac, lung and kidney damage.	[11,43–45,82]<br><br>[23,83–85]
miR-34a	Thrombotic events and Ras signaling<br><br>Can bind to multiple sites on noncoding SARS-CoV-2 RNA, which can affect the host immune response	[19,86]<br><br>[87]
miR-146a	Regulation of cytokine-responsive gene expression, and the pro-inflammatory cytokines like IL-6, TNF-a, IL-1 and IL-8. Regulation of NF-kB, MAP kinase, and STAT 3 pathways. Thrombo-inflammatory processes. Cytokine storm.	[8,13,60,70–72,88,89]
miR-221	Regulation of inflammation and vascular remodeling. Lung injury, coagulopathy, thrombosis.<br><br>Regulation of innate immune response and promoting viral infection.	[19,56,90]<br><br>[44,57–59]

References

[19] C. Eyileten, Z. Wicik, S.N. Simões, D.C. Martins-Jr, K. Klos, W. Wlodarczyk, A. Assinger, D. Soldacki, A. Chcialowski, J.M. Siller-Matula, M. Postula, Thrombosis-related circulating miR-16-5p is associated with disease severity in patients hospitalised for COVID-19, RNA Biol. 19 (2022) 963–979. https://doi.org/10.1080/15476286.2022.2100629.

[77] M. Wang, J. Li, J. Cai, L. Cheng, X. Wang, P. Xu, G. Li, X. Liang, Overexpression of MicroRNA-16 Alleviates Atherosclerosis by Inhibition of Inflammatory Pathways, Biomed Res. Int. 2020 (2020). https://doi.org/10.1155/2020/8504238.

[78] Y. Yang, F. Yang, X. Yu, B. Wang, Y. Yang, X. Zhou, R. Cheng, S. Xia, X. Zhou, miR-16 inhibits NLRP3 inflammasome activation by directly targeting TLR4 in acute lung injury, Biomed. Pharmacother. 112 (2019) 108664. https://doi.org/10.1016/j.biopha.2019.108664.

[79] P. Möhnle, S. Hirschberger, L.C. Hinske, J. Briegel, M. Hübner, S. Weis, G. Dimopoulos, M. Bauer, E.J. Giamarellos-Bourboulis, S. Kreth, MicroRNAs 143 and 150 in whole blood enable detection of T-cell immunoparalysis in sepsis, Mol. Med. 24 (2018) 54. https://doi.org/10.1186/s10020-018-0056-z.

[80] S. Jafarinejad-Farsangi, M.M. Jazi, F. Rostamzadeh, M. Hadizadeh, High affinity of host human microRNAs to SARS-CoV-2 genome: An in silico analysis, Non-Coding RNA Res. 5 (2020) 222–231. https://doi.org/10.1016/j.ncrna.2020.11.005.

[57] W.R. Kim, E.G. Park, K.-W. Kang, S.-M. Lee, B. Kim, H.-S. Kim, Expression Analyses of MicroRNAs in Hamster Lung Tissues Infected by SARS-CoV-2, Mol. Cells. 43 (2020) 953–963. https://doi.org/10.14348/molcells.2020.0177.

[44] M. Molinero, I.D. Benítez, J. González, C. Gort-Paniello, A. Moncusí-Moix, F. Rodríguez-Jara, M.C. García-Hidalgo, G. Torres, J.J. Vengoechea, S. Gómez, R. Cabo, J. Caballero, J.F. Bermejo-Martin, A. Ceccato, L. Fernández-Barat, R. Ferrer, D. Garcia-Gasulla, R. Menéndez, A. Motos, O. Peñuelas, J. Riera, A. Torres, F. Barbé, D. de Gonzalo-Calvo, Bronchial Aspirate-Based Profiling Identifies MicroRNA Signatures Associated With COVID-19 and Fatal Disease in Critically Ill Patients, Front. Med. 8 (2022) 1–15. https://doi.org/10.3389/fmed.2021.756517.

[35] A. Herrera-Van Oostdam, J. Toro-Ortíz, J. López, D. Noyola, D. García-López, N. Durán-Figueroa, E. Martínez-Martínez, D. Portales-Pérez, M. Salgado-Bustamante, Y. López-Hernández, Placental exosomes isolated from urine of patients with gestational diabetes exhibit a differential profile expression of microRNAs across gestation, Int. J. Mol. Med. 46 (2020) 546–560. https://doi.org/10.3892/ijmm.2020.4626.

[81] Z. Wicik, C. Eyileten, D. Jakubik, S.N. Simões, D.C. Martins, R. Pavão, J.M. Siller-Matula, M. Postula, ACE2 Interaction Networks in COVID-19: A Physiological Framework for Prediction of Outcome in Patients with Cardiovascular Risk Factors, J. Clin. Med. 9 (2020) 3743. https://doi.org/10.3390/jcm9113743.

[45] E. Wyler, K. Mösbauer, V. Franke, A. Diag, L.T. Gottula, R. Arsiè, F. Klironomos, D. Koppstein, K. Hönzke, S. Ayoub, C. Buccitelli, K. Hoffmann, A. Richter, I. Legnini, A. Ivanov, T. Mari, S. Del Giudice, J. Papies, S. Praktiknjo, T.F. Meyer, M.A. Müller, D. Niemeyer, A. Hocke, M. Selbach, A. Akalin, N. Rajewsky, C. Drosten, M. Landthaler, Transcriptomic profiling of SARS-CoV-2 infected human cell lines identifies HSP90 as target for COVID-19 therapy, IScience. 24 (2021) 102151. https://doi.org/10.1016/j.isci.2021.102151.

[11] B. Bautista-Becerril, G. Pérez-Dimas, P.C. Sommerhalder-Nava, A. Hanono, J.A. Martínez-Cisneros, B. Zarate-Maldonado, E. Muñoz-Soria, A. Aquino-Gálvez, M. Castillejos-López, A. Juárez-Cisneros, J.S. Lopez-Gonzalez, A. Camarena, miRNAs, from Evolutionary Junk to Possible Prognostic Markers and Therapeutic Targets in COVID-19, Viruses. 14 (2021) 41. https://doi.org/10.3390/v14010041.

[43] R.A.-H. Haroun, W.H. Osman, R.E. Amin, A.K. Hassan, W.S. Abo-Shanab, A.M. Eessa, Circulating plasma miR-155 is a potential biomarker for the detection of SARS-CoV-2 infection, Pathology. 54 (2022) 104–110. https://doi.org/10.1016/j.pathol.2021.09.006.

[82] J. Wang, K. Li, X. Zhang, G. Li, T. Liu, X. Wu, S.L. Brown, L. Zhou, Q.S. Mi, MicroRNA-155 Controls iNKT Cell Development and Lineage Differentiation by Coordinating Multiple Regulating Pathways, Front. Cell Dev. Biol. 8 (2021) 1–18. https://doi.org/10.3389/fcell.2020.619220.

[83] M.G. Katze, Y. He, M. Gale, Viruses and interferon: a fight for supremacy, Nat. Rev. Immunol. 2 (2002) 675–687. https://doi.org/10.1038/nri888.

[84] N. Mitash, J.E. Donovan, A. Swiatecka-Urban, The Role of MicroRNA in the Airway Surface Liquid Homeostasis, Int. J. Mol. Sci. 21 (2020) 3848. https://doi.org/10.3390/ijms21113848.

[85] D.T. Gracias, E. Stelekati, J.L. Hope, A.C. Boesteanu, T.A. Doering, J. Norton, Y.M. Mueller, J.A. Fraietta, E.J. Wherry, M. Turner, P.D. Katsikis, The microRNA miR-155 controls CD8+ T cell responses by regulating interferon signaling, Nat. Immunol. 14 (2013) 593–602. https://doi.org/10.1038/ni.2576.

[23] K. Jiang, J. Yang, S. Guo, G. Zhao, H. Wu, G. Deng, Peripheral Circulating Exosome-Mediated Delivery of miR-155 as a Novel Mechanism for Acute Lung Inflammation, Mol. Ther. 27 (2019) 1758–1771. https://doi.org/10.1016/j.ymthe.2019.07.003.

[86] A. Centa, A.S. Fonseca, S.G. da Silva Ferreira, M.L. V. Azevedo, C.B. V. de Paula, S. Nagashima, C. Machado-Souza, A.F.R. dos Santos Miggiolaro, C. Pellegrino Baena, L. de Noronha, L.R. Cavalli, Deregulated miRNA expression is associated with endothelial dysfunction in post-mortem lung biopsies of COVID-19 patients, Am. J. Physiol. Cell. Mol. Physiol. 320 (2021) L405–L412. https://doi.org/10.1152/ajplung.00457.2020.

[87] R. Bartoszewski, M. Dabrowski, B. Jakiela, S. Matalon, K.S. Harrod, M. Sanak, J.F. Collawn, SARS-CoV-2 may regulate cellular responses through depletion of specific host miRNAs, Am. J. Physiol. Cell. Mol. Physiol. 319 (2020) L444–L455. https://doi.org/10.1152/ajplung.00252.2020.

[88] B.S. Comer, B. Camoretti-Mercado, P.C. Kogut, A.J. Halayko, J. Solway, W.T. Gerthoffer, MicroRNA-146a and microRNA-146b expression and anti-inflammatory function in human airway smooth muscle, Am. J. Physiol. Cell. Mol. Physiol. 307 (2014) L727–L734. https://doi.org/10.1152/ajplung.00174.2014.

[8] D. de Gonzalo-Calvo, I.D. Benítez, L. Pinilla, A. Carratalá, A. Moncusí-Moix, C. Gort-Paniello, M. Molinero, J. González, G. Torres, M. Bernal, S. Pico, R. Almansa, N. Jorge, A. Ortega, E. Bustamante-Munguira, J.M. Gómez, M. González-Rivera, D. Micheloud, P. Ryan, A. Martínez, L. Tamayo, C. Aldecoa, R. Ferrer, A. Ceccato, L. Fernández-Barat, A. Motos, J. Riera, R. Menéndez, D. García-Gasulla, O. Peñuelas, A. Torres, J.F. Bermejo-Martín, F. Barbé, Circulating microRNA profiles predict the severity of COVID-19 in hospitalized patients, Transl. Res. 236 (2021) 147–159. https://doi.org/10.1016/j.trsl.2021.05.004.

[89] A. Anzola, R. González, R. Gámez-Belmonte, B. Ocón, C.J. Aranda, P. Martínez-Moya, R. López-Posadas, C. Hernández-Chirlaque, F. Sánchez de Medina, O. Martínez-Augustin, miR-146a regulates the crosstalk between intestinal epithelial cells, microbial components and inflammatory stimuli, Sci. Rep. 8 (2018) 1–12. https://doi.org/10.1038/s41598-018-35338-y.

[60] J. Sabbatinelli, A. Giuliani, G. Matacchione, S. Latini, N. Laprovitera, G. Pomponio, A. Ferrarini, S. Svegliati Baroni, M. Pavani, M. Moretti, A. Gabrielli, A.D. Procopio, M. Ferracin, M. Bonafè, F. Olivieri, Decreased serum levels of the inflammaging marker miR-146a are associated with clinical non-response to tocilizumab in COVID-19 patients, Mech. Ageing Dev. 193 (2021) 111413. https://doi.org/10.1016/j.mad.2020.111413.

[70] Ana B. Arroyo, María P. Fernández-Pérez, Alberto del Monte, Sonia Águila, Raúl Méndez, Rebecca Hernández-Antolín, Nuria García-Barber, Ascensión M de los Reyes-García, Paula González-Jiménez, María I. Arcas, Vicente Vicente, Rosario Menéndez, Vicente Andrés,

Rocío González-Conejero, Constantino Martínez, miR-146a is a pivotal regulator of neutrophil extracellular trap formation promoting thrombosis, Haematologica. 106 (2020) 1636–1646. https://doi.org/10.3324/haematol.2019.240226.

[13] K.D. Taganov, M.P. Boldin, K.-J. Chang, D. Baltimore, NF-κB-dependent induction of microRNA miR-146, an inhibitor targeted to signaling proteins of innate immune responses, Proc. Natl. Acad. Sci. 103 (2006) 12481–12486. https://doi.org/10.1073/pnas.0605298103.

[71] R. Saba, D.L. Sorensen, S.A. Booth, MicroRNA-146a: A Dominant, Negative Regulator of the Innate Immune Response, Front. Immunol. 5 (2014) 1–11. https://doi.org/10.3389/fimmu.2014.00578.

[72] M.P. Boldin, K.D. Taganov, D.S. Rao, L. Yang, J.L. Zhao, M. Kalwani, Y. Garcia-Flores, M. Luong, A. Devrekanli, J. Xu, G. Sun, J. Tay, P.S. Linsley, D. Baltimore, miR-146a is a significant brake on autoimmunity, myeloproliferation, and cancer in mice, J. Exp. Med. 208 (2011) 1189–1201. https://doi.org/10.1084/jem.20101823.

[90] S. Bátkai, T. Thum, MicroRNAs in Hypertension: Mechanisms and Therapeutic Targets, Curr. Hypertens. Rep. 14 (2012) 79–87. https://doi.org/10.1007/s11906-011-0235-6.

[56] M. Ackermann, S.E. Verleden, M. Kuehnel, A. Haverich, T. Welte, F. Laenger, A. Vanstapel, C. Werlein, H. Stark, A. Tzankov, W.W. Li, V.W. Li, S.J. Mentzer, D. Jonigk, Pulmonary Vascular Endothelialitis, Thrombosis, and Angiogenesis in Covid-19, N. Engl. J. Med. 383 (2020) 120–128. https://doi.org/10.1056/NEJMoa2015432.

[58] P. Pineau, S. Volinia, K. McJunkin, A. Marchio, C. Battiston, B. Terris, V. Mazzaferro, S.W. Lowe, C.M. Croce, A. Dejean, miR-221 overexpression contributes to liver tumorigenesis, Proc. Natl. Acad. Sci. 107 (2010) 264–269. https://doi.org/10.1073/pnas.0907904107.

[59] W.-X. Chen, Q. Hu, M.-T. Qiu, S.-L. Zhong, J.-J. Xu, J.-H. Tang, J.-H. Zhao, miR-221/222: promising biomarkers for breast cancer, Tumor Biol. 34 (2013) 1361–1370. https://doi.org/10.1007/s13277-013-0750-y.
