# Supplementary material for: miR-146a, miR-221, and miR-155 are Involved in Inflammatory Immune Response in Severe COVID-19 Patients
Source: Diagnostics (Basel). 2022 Dec 30;13(1):133. doi: 10.3390/diagnostics13010133 (PMC9818442; doi:10.3390/diagnostics13010133)
Supplement: Supplementary file 1 [file diagnostics-13-00133-s001.zip › diagnostics-2070605-supplementary - final.pdf]

Supplementary Table 1. miRNAs selection criteria

miRNAs	Justification for validation	Reference
miR-16	Involved in regulation of inflammation and programmed cell death, acute lung injury, sepsis, phenotypic changes on T cells survival, differentiation, and proliferation.<br><br>Targeting genes responsible for host-SARS-CoV-2 interaction. Influence viral entry receptor (ACE2) related networks	[19,35,44,57,77–80]<br><br>[57,80,81]
miR-155	Involved in inflammation. T cell differentiation and innate immunity.<br><br>Regulates pathways related with IFN superfamily, NF-kB and MAP Kinase pathways. Cardiac, lung and kidney damage.	[11,43–45,82]<br><br>[23,83–85]
miR-34a	Thrombotic events and Ras signaling<br><br>Can bind to multiple sites on noncoding SARS-CoV-2 RNA, which can affect the host immune response	[19,86]<br><br>[87]
miR-146a	Regulation of cytokine-responsive gene expression, and the pro-inflammatory cytokines like IL-6, TNF-a, IL-1 and IL-8. Regulation of NF-kB, MAP kinase, and STAT 3 pathways. Thrombo-inflammatory processes. Cytokine storm.	[8,13,60,70–72,88,89]
miR-221	Regulation of inflammation and vascular remodeling. Lung injury, coagulopathy, thrombosis.<br><br>Regulation of innate immune response and promoting viral infection.	[19,56,90]<br><br>[44,57–59]
